# Self-sampling for cervical screening offered at the point of invitation: A cross-sectional study of preferences in England

**DOI:** 10.1177/09691413221092246

**Published:** 2022-04-07

**Authors:** Hannah Drysdale, Laura AV Marlow, Anita Lim, Peter Sasieni, Jo Waller

**Affiliations:** 1 Cancer Prevention Group, School of Cancer and Pharmaceutical Sciences, 4616 King’s College London, London, UK

**Keywords:** Self-sampling, cervical screening, human papillomavirus, screening choice, non-attenders, attenders, population survey, healthcare disparities, social class

## Abstract

**Objectives:**

This study assessed preferences for human papillomavirus (HPV) self-sampling if offered as an alternative to clinician-based screening at the point of invitation for cervical screening.

**Setting and Methods:**

An online questionnaire was completed by screening-eligible women living in England (n = 3672). Logistic regressions explored associations between demographic characteristics and screening preferences, stratified by previous screening attendance. Reasons for preferences were also assessed.

**Results:**

Half of participants (51.4%) intended to choose self-sampling, 36.5% preferred clinician screening, 10.5% were unsure, and <2% preferred no screening. More irregular and never attenders chose self-sampling, compared with regular attenders (71.1% and 70.1% vs. 41.0% respectively). Among regular attenders, self-sampling was preferred more frequently by the highest occupational grade, older and lesbian, gay and bisexual women, and those with experience of blood self-tests. In the irregular attender group, older women and those with experience of blood self-tests were more likely to choose self-sampling. In ‘never attenders’, self-sampling was less popular in ethnic minority groups.

**Conclusions:**

If offered a choice of screening, around half of women in England may choose self-sampling, but a substantial proportion would still opt for clinician screening. Screening providers will need to manage a high take-up of self-sampling if many regular attenders switch to self-sampling.

## Introduction

In England, women aged 25–64 years are invited for HPV-based cervical screening. The primary screen used by the NHS Cervical Screening Programme switched from liquid-based cytology to human papillomavirus (HPV) testing in late 2019. HPV-positive samples are processed for cytology-based triage. England has one of the highest cervical screening coverage rates in Europe, but coverage has been declining for ∼20 years.^1^ In 2019–20 coverage was 72.2%^2^ and has likely fallen due to disruption caused by the Covid-19 pandemic.^3^

Barriers to cervical screening are well documented. Embarrassment, fear of pain, discomfort and shame^4–6^ are cited as emotional obstacles to uptake. Childcare responsibilities, inconvenient appointment times, and conflicting priorities can be practical barriers to attendance.^5–7^ HPV testing of self-collected samples has the potential to overcome several types of barriers^8^ and may help to reduce inequalities in cervical screening uptake in specific groups of individuals (e.g. transgender men and women who have experienced domestic violence).^9,10^ Self-sampling is accurate at identifying high-risk HPV;^11^ and can effectively detect CIN2+ (cervical intraepithelial neoplasia grade 2 or worse).^12,13^ Evidence from several countries suggests self-sampling could be an effective way of increasing cervical screening uptake within an organised programme.^11,14^

There are two approaches to offering self-sampling in the context of organised screening programmes. The first is to target screening non-attenders. Studies show that when self-sampling is offered to non-attenders there is an increase in screening uptake (6.4%–34.0% for kits mailed to women; 5.2%–10.9% for opt-in strategies where women request a kit).^11^ However, uptake varies across settings and has been lower in the UK (<10%)^15,16^ compared to Scandinavian countries (∼30%).^12^ Australia, Denmark and the Netherlands^17–19^ now offer self-sampling to non-attenders in their national screening programmes and this approach is currently being trialled in England.^20^ Another strategy would be to offer a choice of self-sampling or clinician sampling to all screening-eligible individuals at the point of invitation. This is being considered in the UK^21^ and will be introduced in Australia in 2022.^22^ However, concerns have been raised about regular screening attenders ‘switching’ to self-sampling which could have an impact on the test's sensitivity to detect CIN2 + if attendance for follow-up cytology or colposcopy is sub-optimal in HPV-positive women.^23^

Little is known about intentions to choose self-sampling if it is offered as a choice at the point of invitation. This information will be vital to planning any choice-based screening offer. Health behaviour models such as the Health Action Process Approach differentiate between motivational (intention formation) and volitional (action) stages of behaviour.^24^ This study assessed intentions (motivational stage) to provide an upper estimate of how many women might select self-sampling over clinician screening if they were offered a choice. We also examined associations between demographic factors and past screening behaviours and preferences for self-sampling, as well as reasons for women's choices.

## Methods

### Design

A cross-sectional questionnaire was hosted online in April 2021. The survey, study protocol, and pre-registered analysis plan are available on Open Science Framework (OSF) (https://osf.io/c54e8/). Ethical approval was granted by the King's College London Research Ethics Committee (LRS-20/21-21776; MOD-20/21-21776).

### Participants

Participants were recruited from an online research panel (Dynata Global UK Ltd). Participants on the online panel sign up to take part in research and are recruited through banner advertising displays and affiliate networks. Participants receive points for completing surveys, which can be redeemed against gift cards or magazine subscriptions. For the present study an email invitation to a ‘cervical screening survey’, including a hyperlink to the questionnaire, was sent.

Screening-eligible women aged 25–64 years living in England were able to take part. Women with a history of cervical cancer or reporting a hysterectomy were ineligible. Quotas were set to ensure the sample was broadly representative of the English population with respect to age (25–44-year-olds; 45–64-year-olds), socioeconomic status (using occupational social grades of ABC1 (high) and C2DE (low))^25^ and region (North; South; Midlands and East). Occupational social grade was used as a marker of socioeconomic status based on the occupation of the Chief Income Earner of the household. Participants allocated themselves to one of six categories (A/B/C1/C2/D/E) based on brief descriptions of the types of occupation included in each.

It was estimated that ∼50% of individuals would select self-sampling. This was a conservative calculation based on findings from other studies.^26,27^ In order to estimate a preference for self-sampling with a 95% confidence interval of ± 3%, 3632 participants were required after adjusting for imbalanced group sizes.

### Measures

Four Public and Patient Involvement (PPI) representatives reviewed the questionnaire to identify areas of the survey that were ambiguous or unclear, and the wording was adjusted accordingly.

*Screening preference*: The primary outcome was women's anticipated choice of screening, after exposure to information on self-sampling and clinician screening. Information was taken from the NHS cervical screening leaflet^28^ and materials developed for a self-sampling trial in England^20^ and explained the similarities and differences between methods. Response options were: ‘Screening done in the same way as now by a nurse or doctor’ (*clinician screening)*, ‘Self-sampling kit to do at home’ (*self-sampling),* ‘I don’t know which screening option I would choose’ (*don’t know)* or ‘Not applicable – I wouldn’t have any cervical screening at all’ (*no screening)*.

*Screening history and intentions*: Participants were asked about their previous screening attendance and were classified as ‘*regular attenders*’ (always attend when invited), ‘*irregular attenders*’ (attended before but sometimes delayed or missed a screening appointment), ‘*never attenders*’ (invited but never attended screening) or ‘*never invited*’ (never been invited for screening). Women were asked about their intention to attend screening the next time they were invited (yes, definitely; yes, probably; no, probably not; no, definitely not). We also asked about previous experiences of a follow-up to screening and experience of other self-tests.

*Factors influencing women's choice of screening*: Facilitators and barriers to clinician screening^4–6,29,30^ and self-sampling^26,31,32^ were taken from the literature. Different factors were presented (response options: “this affected my choice” or “this did not affect my choice”). There was also a free text response option to report other factors. A coding frame was developed for the free-text responses and two members of the research team independently coded the data (Cohen's Kappa = 84% for self-sampling and 82% for clinician screening). Discrepancies were resolved through discussion.

*Demographic information*: We collected information on socioeconomic status (occupational social grade), age, region, marital status, sexual orientation, ethnicity, and HPV vaccination status. Ethnicity and marital status categories were collapsed for analysis. Sexual orientation was recoded into heterosexual and lesbian, gay, bisexual and other (referred to as “LGB”).

### Analysis

Analyses were conducted using IBM SPSS Statistics Version 26.0. Two attention check questions were used to exclude individuals who had not read the questions properly. Women were also excluded if they completed the survey too quickly (<120 s) or slowly (>3 standard deviations above the mean time).

The proportion of women who selected each screening option was reported overall and by screening history and demographic factors.

Binary logistic regression analyses explored the association between demographics and screening choice. The planned analysis (Table S1, Supplementary materials) identified previous screening attendance as the most significant predictor of screening choice. Therefore, we stratified the analysis to understand the effect of other predictor variables within the different screening status groups (‘regular attenders’, ‘irregular attenders’, ‘never attenders’).

An a priori decision was made to include age, ethnicity, sexual orientation, marital status and socioeconomic status in the models as they are established predictors of screening behaviours. Other variables were included if p < 0.1 in univariable analysis. HPV vaccination status was not included as most women had not been offered the vaccine (n = 2939/3672; 80.0%). “Prefer not to say” responses were excluded on a pairwise basis. The first regression compared self-sampling with clinician screening (reference group) and the second compared ‘don’t know’ responses with those that made a screening choice (reference group). Never attenders were not included in the second regression due to small group size (n = 26). The group that chose ‘no screening’ (n = 60) and those who had never been invited for screening (n = 83) were also excluded.

## Results

### Participant characteristics

A total of 5868 women started the survey; 1689 were ineligible and 325 were excluded (failed attention checks or completed the survey too quickly/slowly). Therefore, the results presented are for 3672 women (see Figure S1 in the Supplementary materials for further detail).

Table 1 shows participant characteristics. The median age was 44.0 years with a range of 25–64 years (inter-quartile range: 35.0–53.0). Most women were White British (89.4%), heterosexual (93.3%), and in a relationship (67.1%). Half (50.3%) identified as occupational social grade B (intermediate managerial) or C1 (supervisory, clerical, and junior managerial). Most were self-reported regular screening attenders (63.5%) and 85.4% intended to attend screening the next time they were invited. A quarter reported having a screening result that needed follow-up and 83.8% had experienced self-testing. Only 10.6% had received the HPV vaccination.

**Table 1.** Participant characteristics (n = 3672).

	N	**%**
**Age (median)**	44.0 (Range: 25–64)	
**Region**	** **	
North England	1055	28.7
South England	1531	41.7
The Midlands and East of England	1086	29.6
**Ethnicity**	** **	
Any White background	3284	89.4
Mixed/Multiple background	68	1.9
Asian/Asian British background	207	5.6
Black/African/Caribbean/Black British background	73	2.0
Other Ethnic background	14	0.4
Prefer not to say	26	0.7
**Marital Status**	** **	
Single	840	22.9
Married, civil partnership or cohabiting	2315	63.0
In a relationship but not living together	148	4.0
Divorced or separated	302	8.2
Widowed	51	1.4
Other	3	0.1
Prefer not to say	13	0.4
**Sexual Orientation**	** **	
Heterosexual/Straight	3427	93.3
Gay or Lesbian	79	2.2
Bisexual	106	2.9
Other	20	0.5
Prefer not to say	40	1.1
**HPV Vaccination Status**	** **	
Yes, had the vaccine	389	10.6
No, declined the offer of a vaccine	84	2.3
No, not offered the vaccine	2855	77.7
Don’t know	330	9.0
Prefer not to say	14	0.4
**Education**		
No qualifications	71	1.9
GCSEs or equivalent	893	24.3
AS, A-Level or equivalent	942	25.7
Degree or above	1764	48.0
Prefer not to say	2	0.1
**Occupational social grade**	** **	
A – higher managerial occupations	197	5.4
B – intermediate managerial occupations	802	21.8
C1 – supervisory/junior managerial occupations	1045	28.5
C2 – skilled manual workers	715	19.5
D – semi-skilled and unskilled manual workers	538	14.6
E – state pensioners, low-grade workers, unemployed	375	10.2
**Economic Stress (ability to make ends meet)**	** **	
With great difficulty	217	5.9
With moderate difficulty	332	9.0
With some difficulty	852	23.2
Fairly easily	1178	32.1
Easily	586	16.0
Very easily	431	11.7
Prefer not to say	76	2.1
**Subjective Social Status** (n = 3637; possible range 1–10))	M = 5.3, SD = 1.7	
**Previous cervical screening attendance**		
I have always attended when invited (within 6 months)	2333	63.5
I have attended before but have sometimes delayed or missed my screening appointment	892	24.3
I have been invited but have never attended	351	9.6
I have never been invited for cervical screening	83	2.3
Prefer not to say	13	0.3
**Future screening intentions (will you go when next invited?)**	** **	
Yes, definitely	2586	70.4
Yes, probably	551	15.0
No probably not	261	7.1
No, definitely not	118	3.2
Don't know	151	4.1
Prefer not to say	5	0.2
**Previous follow-up to a cervical screening result** ^A^	** **	
No	2667	72.6
Yes	937	25.5
Don’t know	61	1.7
Prefer not to say	7	0.2
**Previous experience of using self-tests**	** **	
No	591	16.1
Prefer not to say	4	0.1
Yes	3077	83.8
* Pregnancy test* ^B^	2341	63.8
* Urine test* ^B^	628	17.1
* Vaginal swab* ^B^	369	10.0
* Glucose tests for diabetes* ^B^	416	11.3
* Blood test* ^B^	367	10.0
* Covid-19 infection test* ^B^	1528	41.6
* Covid-19 antibody test* ^B^	0	0.0
* Bowel screening test* ^B^	329	9.0
* Other test* ^B^	6	0.2

^A^ Yes responses included: repeat screening because of an inadequate test; screening test sooner than usual; colposcopy at a hospital clinic; biopsy or treatment for abnormal cell; invited for a repeat test but did not attend.

^B^ Percentages are calculated as a proportion of the total sample size (n = 3672).

### Overall choice of screening test

Before information exposure, participants were equally likely to select clinician screening (42.9%) and self-sampling (42.6%), 13.1% selected ‘don’t know’ and 1.4% chose not to be screened. After exposure, there was a shift towards self-sampling: 51.4% chose self-sampling, 36.5% preferred clinician screening, 10.5% selected ‘don’t know’ and 1.6% still opted for no screening (Table 2). Post-information choices by demographic group, screening status and self-test experience are shown in Table 2.

**Table 2.** Women's preferences for each screening option after information exposure.

	Proportion selecting each screening option (row %, 95% CI)			
	Clinician Screening (n = 1340)	Self-sampling (n = 1886)	Don't know (n = 386)	No screening (n = 60)
**Overall**	36.5 (34.9–38.1)	51.4 (49.7–53.0)	10.5 (9.5–11.6)	1.6 (1.3–2.1)
**Age (mean; standard deviation)**	M = 43.1, SD = 11.1	M = 44.7, SD = 11.0	M = 44.0, SD = 10.4	M = 45.4, SD = 13.1
**Ethnicity**				
Any White background (n = 3284)	36.2 (34.5–37.9)	51.9 (50.2–53.6)	10.4 (9.4–11.5)	1.5 (1.1–2.0)
Ethnic minority background (n = 362)	39.8 (34.7–45.0)	46.4 (41.2–51.7)	11.0 (8.0–14.7)	2.8 (1.3–5.0)
**Marital Status**				
Single (n = 840)	34.1 (30.8–37.4)	52.1 (48.7–55.6)	10.2 (8.3–12.5)	3.6 (2.4–5.1)
Separated, Divorced, Widowed (n = 353)	35.1 (30.2- 40.4)	54.1 (48.8–59.4)	8.8 (6.0–12.2)	2.0 (0.8–4.0)
In a relationship (n = 2463)	37.5 (35.6–39.4)	50.8 (48.8–52.8)	10.8 (9.6–12.1)	0.9 (0.6–1.4)
**Sexual Orientation**				
Heterosexual (n = 3427)	37.2 (35.6–38.9)	50.8 (49.1–52.5)	10.6 (9.6–11.6)	1.4 (1.0–1.8)
Gay, Lesbian, Bisexual or Other (n = 205)	26.3 (20.5–32.9)	59.5 (52.5–66.3)	9.8 (6.1–14.7)	4.4 (2.0–8.2)
**Region**				
North England (n = 1055)	39.8 (36.8–42.8)	48.2 (45.1–51.2)	10.5 (8.7–12.5)	1.5 (0.9–2.5)
South England (n = 1531)	35.9 (33.5–38.3)	50.8 (48.3–53.4)	11.3 (9.8–13.0)	2.0 (1.4–2.9)
The Midlands and East England (n = 1086)	34.2 (31.3–37.1)	55.2 (52.3–58.2)	9.4 (7.7–11.3)	1.2 (0.6–2.0)
**Occupational social grade**				
DE (lowest social grade) (n = 913)	34.9 (31.9–38.1)	51.4 (48.1–54.7)	11.3 (9.3–13.5)	2.4 (1.5–3.6)
C2 (n = 715)	41.8 (38.2–45.5)	45.3 (41.6–49.1)	11.8 (9.5–14.3)	1.1 (0.5–2.2)
C1 (n = 1045)	36.5 (33.5–39.5)	52.6 (49.6–55.7)	9.6 (7.9–11.5)	1.3 (0.7–2.2)
AB (highest social grade) (n = 999)	34.1 (31.2–37.2)	54.4 (51.2–57.5)	9.9 (8.1–11.9)	1.6 (0.9–2.6)
**Education**				
GCSEs and below (n = 964)	35.9 (32.9–39.0)	51.8 (48.6–55.0)	11.0 (9.1–13.1)	1.3 (0.7–2.3)
A-Levels or equivalent (n = 942)	36.0 (32.9–39.2)	51.3 (48.0–54.5)	10.9 (9.1–13.1)	1.8 (1.1–2.9)
Degree and above (n = 1764)	37.0 (34.8–39.3)	51.3 (48.9–53.6)	10.0 (8.7–11.5)	1.7 (1.2–2.4)
**HPV Vaccination Status**				
Vaccinated (n = 389)	43.4 (38.5–48.5)	47.3 (42.3–52.4)	8.5 (5.9–11.7)	0.8 (0.2–2.2)
Not offered the vaccine (n = 2855)	34.6 (32.9–36.4)	53.0 (51.1–54.8)	10.7 (9.6–12.0)	1.7 (1.2–2.2)
Declined the offer of a vaccine (n = 84)	44.0 (33.2–55.3)	42.9 (32.1–54.1)	9.5 (4.2–17.9)	3.6 (0.7–10.1)
Don’t know (n = 330)	42.4 (37.0–48.0)	44.6 (39.1–50.1)	11.2 (8.0–15.1)	1.8 (0.7–3.9)
**Previous screening attendance**				
Regular attenders (n = 2333)	46.8 (44.8–48.9)	41.0 (39.0–43.1)	11.7 (10.4–13.0)	0.5 (0.2–0.8)
Irregular attenders (n = 892)	18.7 (16.2–21.4)	71.1 (68.0–74.0)	9.0 (7.2–11.0)	1.2 (0.6–2.2)
Never attenders (n = 351)	12.5 (9.3–16.5)	70.1 (65.0–74.8)	7.4 (4.9–10.7)	10.0 (7.0–13.6)
Never been invited (n = 83)	41.0 (30.3–52.3)	49.4 (38.2–60.6)	7.2 (2.7–15.1)	2.4 (0.3–8.4)
**Follow-up to a previous screening**				
Yes (n = 937)	43.0 (39.8–46.3)	45.9 (42.7–49.1)	10.8 (8.9–12.9)	0.3 (0.1–0.9)
No (n = 2667)	34.1 (32.3–35.9)	53.5 (51.6–55.5)	10.4 (9.3–11.6)	2.0 (1.5–2.6)
**Future screening intentions**				
Yes, definitely attend (n = 2586)	46.6 (44.7–48.5)	42.2 (40.2–44.1)	10.9 (9.7–12.2)	0.3 (0.2–0.7)
Yes, probably attend (n = 551)	20.3 (17.0–23.9)	67.9 (63.8–71.8)	11.1 (8.6–14.0)	0.7 (0.2–1.9)
No, probably not attend (n = 261)	2.7 (1.1–5.5)	84.3 (79.3–88.5)	8.8 (5.7–12.9)	4.2 (2.1–7.4)
No, definitely not attend (n = 118)	2.5 (0.5–7.3)	67.0 (57.7–75.3)	4.2 (1.4–9.6)	26.3 (18.6–35.2)
**Experience of using any self-tests**				
Not used a self-test (n = 591)	38.9 (35.0–43.0)	46.7 (42.6–50.8)	10.0 (7.7–12.7)	4.4 (2.9–6.4)
Used a self-test (n = 3081)	36.0 (34.3–37.8)	52.3 (50.5–54.0)	10.6 (9.6–11.8)	1.1 (0.8–1.5)
***Pregnancy test***				
Not used the test (n = 1331)	34.3 (31.7–36.9)	52.7 (50.0–55.5)	10.1 (8.5–11.8)	2.9 (2.1–4.0)
Used the test (n = 2341)	37.8 (35.8–39.8)	50.6 (48.5–52.6)	10.7 (9.5–12.1)	0.9 (0.6–1.4)
***Urine test***	*** ***			
Not used the test (n = 3044)	36.5 (34.8–38.3)	50.4 (48.6–52.2)	11.2 (10.1–12.3)	1.9 (1.4–2.4)
Used the test (n = 628)	36.3 (32.5–40.2)	55.9 (51.9–59.8)	7.3 (5.4–9.7)	0.5 (0.1–1.4)
***Vaginal swab***				
Not used the test (n = 3303)	35.5 (33.9–37.2)	51.9 (50.2–53.7)	10.9 (9.8–12.0)	1.7 (1.3–2.2)
Used the test (n = 369)	45.3 (40.1–50.5)	46.1 (40.9–51.3)	7.3 (4.9–10.5)	1.3 (0.4–3.1)
***Blood test (Glucose, Covid-19 antibody)***				
Not used the test (n = 2967)	37.3 (35.6–39.1)	50.5 (48.6–52.3)	10.5 (9.4–11.6)	1.7 (1.3–2.3)
Used the test (n = 705)	32.9 (29.5–36.5)	55.2 (51.4–58.9)	10.6 (8.5–13.2)	1.3 (0.6–2.4)
***Covid infection test***				
Not used the test (n = 2144)	37.0 (35.0–39.1)	50.2 (48.0–52.3)	10.3 (9.1–11.7)	2.5 (1.9–3.3)
Used the test (n = 1528)	35.7 (33.3–38.2)	53.1 (50.5–55.6)	10.8 (9.3–12.5)	0.4 (0.1–0.9)
***Bowel cancer screening test***				
Not used the test (n = 3343)	36.7 (35.1–38.4)	50.9 (49.2–52.6)	10.8 (9.7–11.9)	1.6 (1.2–2.1)
Used the test (n = 329)	34.1 (28.9–39.4)	56.2 (50.7–61.7)	7.9 (5.2–11.4)	1.8 (0.7–3.9)

### Self-sampling choice versus clinician screening (please see Table S2, supplementary materials)

For all three screening groups, the regression models were statistically significant (p < .05).

*Regular attenders*: The odds of choosing self-sampling compared with clinician screening increased with age (AOR = 1.02 (95%CI: 1.01–1.03) per year of age) and were higher for LGB compared with heterosexual women (AOR = 2.16 (95%CI: 1.29–3.63)). The odds of selecting self-sampling were higher for women who had experience of using blood self-tests (AOR = 1.38 (95%CI: 1.07–1.77)) than those without and were greater for women in the highest occupational social grade (AB vs. DE) (AOR = 1.37 (95%CI: 1.03–1.82)).

*Irregular attenders:* The odds of choosing self-sampling compared with clinician screening increased with age (AOR = 1.04 (95%CI: 1.01–1.06) per year of age) and were greater for women who had experience of using blood self-tests (AOR = 2.17 (95%CI: 1.17–4.03)) than those without.

*Never attenders*: The odds of choosing self-sampling compared with clinician screening were lower for women from ethnic minority backgrounds (AOR = 0.22 (95%CI: 0.08–0.59)), compared with women from a white background.

### Don’t know response versus making a choice (please see Table S3, supplementary materials)

Both regression models were statistically significant (p < .05).

*Regular attenders*: The odds of selecting a ‘don’t know’ response compared with making a choice was lower for those who had experience of using urine self-tests (AOR = 0.49 (95%CI: 0.31–0.79)) than those without, and for women who were separated, divorced or widowed (AOR = 0.51 (95%CI: 0.27–0.95)), compared with those in a relationship.

*Irregular attenders:* The odds of selecting a ‘don’t know’ response compared with making a choice was greater for women from ethnic minority backgrounds (AOR = 2.18 (95%CI: 1.02–4.67)), compared with women from a white background; and were higher for separated, divorced or widowed women (AOR = 2.65 (95%CI: 1.30–5.40)) than those in a relationship.

### Reasons for screening choice (*Figure 1*)

The most frequently endorsed reasons for selecting self-sampling included ease (81.9%; n = 1545/1886), comfort (79.3%), privacy (77.7%), convenience (74.5%) and reduced embarrassment (69.5%). Women who preferred clinician screening had more confidence in the test being done correctly (85.7%; n = 1148/1340), greater trust in the results (84.9%) and preferred the status quo (70.5%). About a third of women (n = 1169) reported other factors influencing their screening choice (Tables S4 and S5 in Supplementary materials). Previous negative experiences with clinicians or the screening procedure and a preference for non-speculum sampling methods were the most cited reasons for choosing self-sampling. A lack of confidence in their ability to do the test and screening being seen as a job for professionals were the most cited reasons for choosing clinician screening.

**Figure 1.**
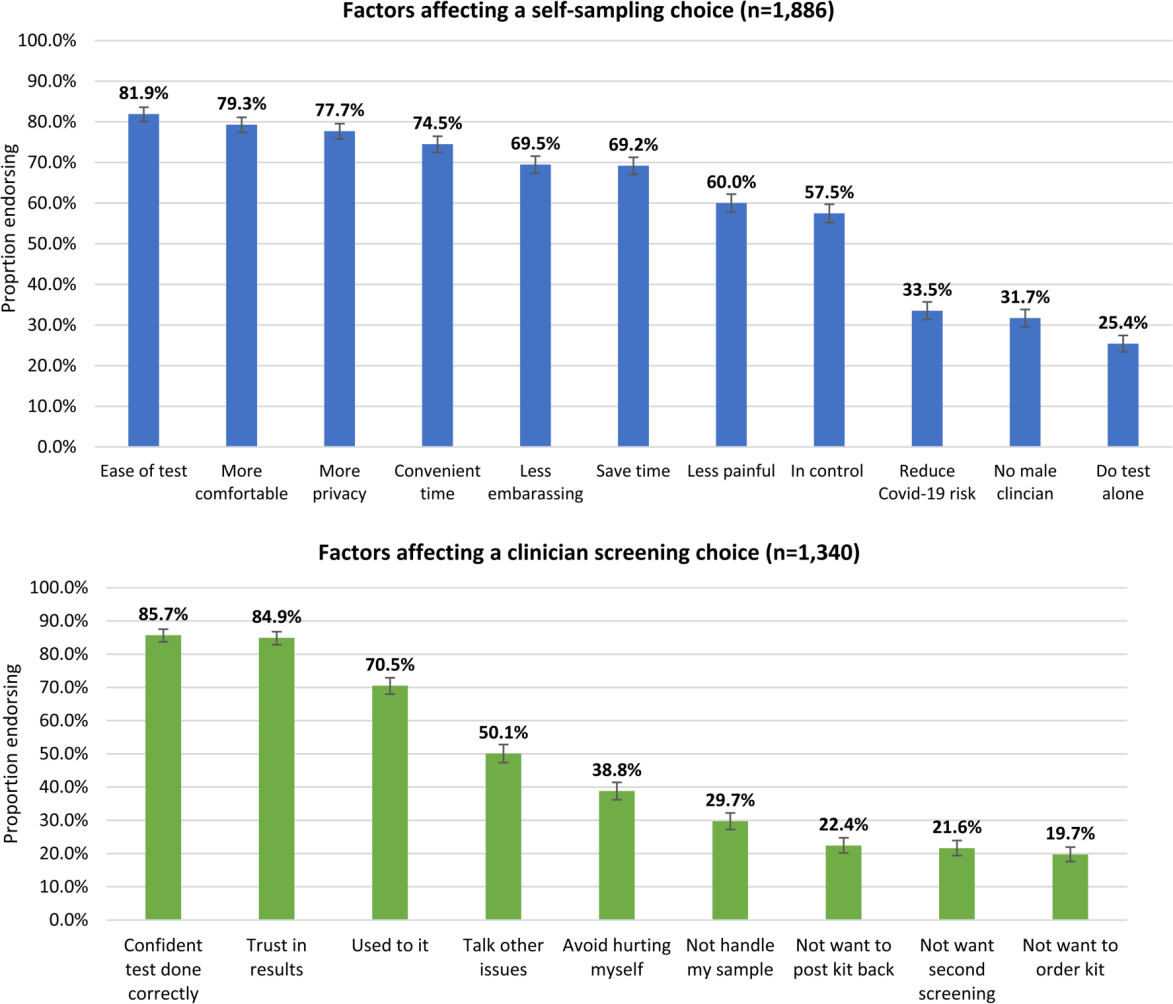
Factors affecting women’s screening choice.

### Engagement with self-sampling among non-intenders

At the start of the survey, 10.3% of women (n = 379) said that they did not intend to go to screening in the future (“non-intenders”). However, after they had been presented with further information, 78.9% of non-intenders (n = 299/379) said that they would choose self-sampling in the future.

## Discussion

This is the first study to consider women's anticipated preferences for self-sampling if they were offered a screening choice at the point of invitation. We found that in a group of women, most of whom reported regularly attending cervical screening, around half anticipate choosing self-sampling, but over a third would still prefer clinician screening. The results support existing literature showing that self-sampling is highly acceptable and is consistent with a systematic review that calculated a pooled estimate of 59% of women preferring self-sampling.^26^ A key difference is that most studies included in the review focused on screening non-attenders. Our research also shows that offering women a choice could help increase the overall proportion who would choose to be screened. Therefore, we think it is reasonable to expect that the introduction of self-sampling, combined with offering a choice, may increase coverage in the future.

The proportion of women selecting self-sampling was higher for irregular attenders, in line with the literature^12,29^ and our results showed a similar effect for never attenders. However, the ‘intention-behaviour gap’ is well-recognised.^33^ A colorectal cancer study examining the gap between screening intentions (*motivation)* and attendance (*action)* found only 72% of those who said they would ‘definitely attend’ actually participated in screening. This intention-behaviour gap is also present in the context of self-sampling, with uptake among non-attenders being lower (<10%)^15,16^ than preferences for self-sampling would indicate (59%).^26^ Our findings demonstrate that the *motivation* to take part in screening may be higher than previously thought in non-attenders when they are offered a choice. This highlights the importance of volition-based interventions to translate self-sampling intentions into behaviour. Text message reminders and planning self-regulatory micro-actions (e.g. putting the kit in the bathroom) may help facilitate uptake.^34,35^

Demographic differences in self-sampling preferences were identified. Among regular and irregular attenders, self-sampling preferences increased with age. One explanation is that older women tend to find the speculum more painful post-menopause and prefer non-speculum methods.^4^ A recent trial found that offering older women a choice of self-sampling or non-speculum clinician screening increased uptake over 12 months by 17%.^36^ In regular attenders, LGB women had greater odds of choosing self-sampling. There was no such association in irregular and never attenders, suggesting self-sampling may not address certain barriers to cervical screening in LGB women who do not currently attend, such as the perception of being low-risk or ineligible.^37^ Although there were socioeconomic status differences in self-sampling preferences in regular attenders, this was not apparent for irregular and never attenders. However, implementation studies, such as YouScreen,^20^ which has offered women in London the opportunity to take a self-sample and has integrated it into the NHS Cervical Screening Programme for the first time, will be key to understanding any impact of self-sampling on inequalities in cervical screening uptake. Finally, in never attenders, women from ethnic minority backgrounds were less likely to choose self-sampling than white women. Further research should explore the impact of offering a screening choice on existing ethnic disparities in screening attendance.^38^

Our findings suggest a considerable proportion of regular screeners might switch to self-sampling if offered a choice. This may have implications for cervical screening programmes in the future. One benefit may be less demand for primary care services, which could free up resources. However, additional infrastructure will be required, and dry self-samples require more manual pre-processing. Further modelling is required to assess the impact of switching behaviours on the performance of established cervical screening programmes. Detection and treatment of CIN2 + could decrease unless there is high compliance with follow-up testing after an HPV-positive self-sampling result.^23^

In this study, there was some evidence that women in the lowest occupational grades (in never attenders) and women from ethnic minority backgrounds (in irregular attenders) were more likely to select a don’t know response. This may suggest additional information needs, to support women in making a choice.

Reasons for self-sampling preference were consistent with previous literature.^4,8,39^ Like other studies, we found women preferring clinician screening lacked confidence in doing the self-sampling test correctly^8,26,29,32,39^ and had less trust in self-sampling results, consistent with experiences of women in the Netherlands.^40^ It is imperative that any roll-out of self-sampling, particularly to non-attenders, tries to address these concerns.

Finally, our results showed that preferences can alter when women are provided with more information on screening. This highlights the importance of clear communication and should be considered by any screening programmes planning to implement novel screening methods.

### Limitations

Individuals from deprived, non-English speaking and ethnic minority groups were under-represented in our study. In addition, we need to caveat our findings for never attenders. While this group were more likely to endorse that they would have “no screening”, a high proportion also endorsed that they would self-sample or have clinician screening. This is at odds with the expected screening intentions of never attenders. Our finding that women from ethnic minority backgrounds were more likely to choose self-sampling than clinician screening is inconsistent with a recent clinical study.^36^ There will likely be heterogeneity in preferences within ethnic minority women and further research is needed to understand how preferences vary between, and within, ethnic subgroups. Finally, we only offered the option of vaginal self-sampling, but urine self-sampling may change women's preferences in the future.^41^

## Conclusions

Our findings suggest around half of women in England invited for cervical screening may choose self-sampling if offered a choice, with a significant proportion of regular attenders likely to switch. This potentially high take-up of self-sampling needs to be considered as the implementation of self-sampling into screening programmes is planned. Furthermore, our results suggest that offering a choice of screening may help to increase overall screening participation.

## Supplemental Material

sj-docx-1-msc-10.1177_09691413221092246 - Supplemental material for Self-sampling for cervical screening offered at the point of invitation: A cross-sectional study of preferences in England Supplemental material, sj-docx-1-msc-10.1177_09691413221092246 for Self-sampling for cervical screening offered at the point of invitation: A cross-sectional study of preferences in England by Hannah Drysdale, Laura AV Marlow, Anita Lim, Peter Sasieni and Jo Waller in Journal of Medical Screening
