# Supplementary material for: Self-sampling for cervical screening offered at the point of invitation: A cross-sectional study of preferences in England
Source: J Med Screen. 2022 Apr 7;29(3):194–202. doi: 10.1177/09691413221092246 (PMC9381689; doi:10.1177/09691413221092246)
Supplement: sj-docx-1-msc-10.1177_09691413221092246 - Supplemental material for Self-sampling for cervical screening offered at the point of invitation: A cross-sectional study of preferences in England [file sj-docx-1-msc-10.1177_09691413221092246.docx]

**Supplementary materials**

*This document provides further detail on eligible, ineligible, and excluded study participants (Figure S1). It contains the planned binary logistic regression analysis which explored the association between demographic factors, screening history, and screening choice; and identified previous screening attendance as the most significant predictor of choice (Table S1). This file also includes the binary logistic regression exploring predictors of a self-sampling choice (compared to clinician screening) stratified by previous screening attendance (Table S2), and the binary logistic regression exploring predictors of a don’t know response (compared to making a screening choice) stratified by previous screening attendance (Table S3). Other reported factors influencing a woman’s choice of screening (coded from free-text responses) are presented in Tables S4 and S5.*

**
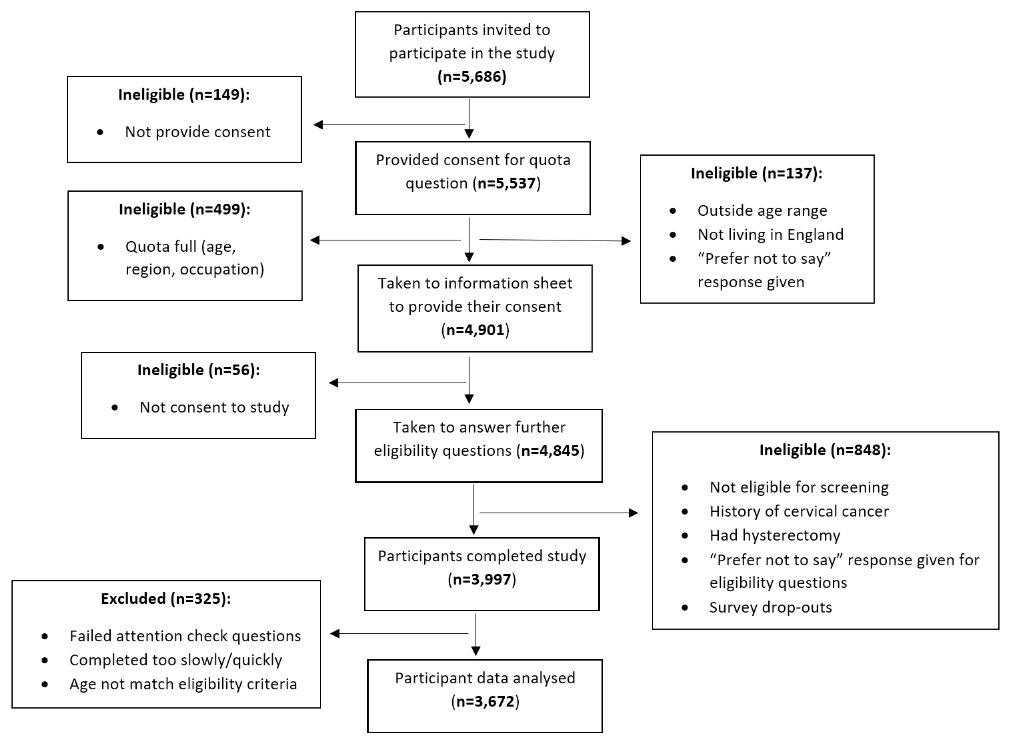
Figure S1: Flowchart of eligible, ineligible, and excluded study participants**

**Table S1: Binary Logistic Regressions exploring predictors of a self-sampling choice (compared to clinician screening) and don’t know responses (compared to making a choice)**

|  | **Self-sampling choice vs clinician screening choice (ref)** | | | | **Don't know response vs making a screening choice (ref)** | | | |
| --- | --- | --- | --- | --- | --- | --- | --- | --- |
|  | *Univariable analysis* | | *Multivariable analysis^a^* | | *Univariable analysis* | | *Multivariable analysis^b^* | |
|  | *OR (95% CI)* | *p* | *AOR (95% CI)* | *p* | *OR (95% CI)* | *p* | *AOR (95% CI)* | *p* |
| **Age (continuous)** | 1.01 (1.00-1.02) | **<.001** | 1.03 (1.02-1.04) | **<.001** | 1.00 (0.99-1.01) | 0.911 | 0.99 (0.98-1.01) | 0.299 |
| **Ethnicity** |  |  |  |  |  |  |  |  |
| Any White background | *Reference* |  |  |  |  |  |  |  |
| Ethnic minority background | 0.81 (0.64-1.03) | 0.084 | 0.79 (0.58-1.08) | 0.136 | 1.09 (0.77-1.54) | 0.649 | 1.23 (0.81-1.86) | 0.329 |
| **Sexual Orientation** |  |  |  |  |  |  |  |  |
| Heterosexual | *Reference* |  |  |  |  |  |  |  |
| Lesbian, Gay, Bisexual and Other (LGB) | 1.66 (1.19-2.30) | **0.003** | 1.97 (1.28-3.04) | **0.002** | 0.95 (0.59-1.52) | 0.824 | 1.19 (0.71-2.00) | 0.516 |
| **Marital Status** |  |  |  |  |  |  |  |  |
| In a relationship | *Reference* |  |  |  |  |  |  |  |
| Single | 1.13 (0.95-1.34) | 0.163 | 0.99 (0.78-1.25) | 0.909 | 0.97 (0.75-1.25) | 0.799 | 0.89 (0.65-1.22) | 0.454 |
| Separated, divorced, widowed | 1.14 (0.89-1.45) | 0.299 | 0.93 (0.69-1.26) | 0.629 | 0.80 (0.54-1.18) | 0.266 | 0.88 (0.57-1.35) | 0.549 |
| **Occupational social grade** |  |  |  |  |  |  |  |  |
| DE (lowest social grade) | *Reference* |  |  |  |  |  |  |  |
| C2 | 0.74 (0.60-0.91) | **0.005** | 0.98 (0.75-1.28) | 0.876 | 1.03 (0.76-1.40) | 0.843 | 0.90 (0.64-1.28) | 0.556 |
| C1 | 0.98 (0.81-1.19) | 0.853 | 1.22 (0.96-1.56) | 0.103 | 0.82 (0.61-1.10) | 0.186 | 0.70 (0.50-0.98) | **0.035** |
| AB (highest social grade) | 1.08 (0.89-1.32) | 0.426 | 1.39(1.08-1.780 | **0.011** | 0.86 (0.64-1.15) | 0.300 | 0.72 (0.51-1.01) | 0.054 |
| **Education** |  |  |  |  |  |  |  |  |
| GCSEs and below | *Reference* |  |  |  |  |  |  |  |
| A-Levels or equivalent | 0.99 (0.81-1.20) | 0.930 |  |  | 1.00 (0.75-1.33) | 0.994 |  |  |
| Degree and above | 0.96 (0.81-1.13) | 0.637 |  |  | 0.91 (0.70-1.17) | 0.449 |  |  |
| **Previous screening attendance** |  |  |  |  |  |  |  |  |
| Attenders | *Reference* |  |  |  |  |  |  |  |
| Non-attenders | 6.39 (4.58-8.90) | **<.001** | 8.53 (5.59-13.02) | **<.001** | 0.68 (0.44-1.03) | 0.068 | 0.69 (0.43-1.12) | 0.131 |
| Delayed attenders | 4.34 (3.58-5.25) | **<.001** | 5.04 (4.01-6.34) | **<.001** | 0.75 (0.58-0.98) | **0.034** | 0.74 (0.55-1.00) | **0.049** |
| **Follow-up to a previous screening** |  |  |  |  |  |  |  |  |
| No | *Reference* |  |  |  |  |  |  |  |
| Yes | 0.68 (0.58 – 0.80) | **<.001** | 0.83 (0.68-1.00) | 0.054 | 1.02 (0.80-1.30) | 0.856 |  |  |
| **Used a pregnancy self-test** |  |  |  |  |  |  |  |  |
| No | Reference |  |  |  |  |  |  |  |
| Yes | 0.87 (0.75-1.01) | 0.063 | 1.06 (0.87-1.29) | 0.549 | 1.05 (0.84-1.31) | 0.647 |  |  |
| **Used a covid-19 infection self-test** |  |  |  |  |  |  |  |  |
| No | Reference |  |  |  |  |  |  |  |
| Yes | 1.10 (0.95-1.27) | 0.201 |  |  | 1.03 (0.83-1.27) | 0.798 |  |  |
| **Used a bowel cancer screening self-test** |  |  |  |  |  |  |  |  |
| No | Reference |  |  |  |  |  |  |  |
| Yes | 1.19 (0.93-1.53) | 0.161 |  |  | 0.71 (0.47-1.08) | 0.109 |  |  |
| **Used a blood self-test** |  |  |  |  |  |  |  |  |
| No | Reference |  |  |  |  |  |  |  |
| Yes | 1.24 (1.04-1.49) | **0.019** | 1.47 (1.17-1.85) | **<.001** | 1.01 (0.78-1.32) | 0.932 |  |  |
| **Used a urine self-test** |  |  |  |  |  |  |  |  |
| No | Reference |  |  |  |  |  |  |  |
| Yes | 1.12 (0.93-1.34) | 0.245 |  |  | 0.62 (0.45-0.85) | **0.003** | 0.54 (0.37-0.80) | **0.002** |
| **Used a vaginal self-test** |  |  |  |  |  |  |  |  |
| No | Reference |  |  |  |  |  |  |  |
| Yes | 0.70 (0.56-0.87) | **0.002** | 0.82 (0.61-1.12) | 0.213 | 0.65 (0.43-0.97) | **0.035** | 0.78 (0.49-1.23) | 0.284 |

*Note: Only variables that were p<.1 in the univariable analysis or an a-priori decision had been made to include them in the multivariable models are included in the table.*

^a^ Pseudo R² = 0.19; ^b^ Pseudo R² = 0.02

**Table S2: Binary Logistic Regression exploring predictors of a self-sampling choice (compared to clinician screening), stratified by previous screening attendance**

|  | ***Regular Attenders (n=1,657)*** | | | ***Irregular Attenders (n=635)*** | | | ***Never attenders (n=222)*** | | |
| --- | --- | --- | --- | --- | --- | --- | --- | --- | --- |
|  | *% choosing self-sampling* | *AOR (95% CI)* | *p* | *% choosing self-sampling* | *AOR (95% CI)* | *p* | *% choosing self-sampling* | *AOR (95% CI)* | *p* |
| **Age (continuous)** |  | 1.02 (1.01-1.03) | **<0.001** |  | 1.04 (1.01-1.06) | **0.003** |  | 1.05 (1.00-1.10) | 0.079 |
| **Ethnicity** |  |  |  |  |  |  |  |  |  |
| Any white background (Ref) | 46.8 |  |  | 80.1 |  |  | 89.3 |  |  |
| Ethnic minority background | 44.4 | 0.98 (0.68 – 1.40) | 0.902 | 69.8 | 0.76 (0.37-1.54) | 0.441 | 65.4 | 0.22 (0.08 -0.59) | **0.003** |
| **Sexual Orientation** |  |  |  |  |  |  |  |  |  |
| Heterosexual (Ref) | 46.2 |  |  | 79.2 |  |  | 83.7 |  |  |
| Lesbian, Gay, Bisexual and Other (LGB) | 57.0 | 2.16 (1.29-3.63) | **0.004** | 77.6 | 1.18 (0.49-2.83) | 0.719 | 88.9 | 2.21 (0.45-10.74) | 0.327 |
| **Marital Status** |  |  |  |  |  |  |  |  |  |
| In a relationship (Ref) | 47.4 |  |  | 80.4 |  |  | 82.7 |  |  |
| Single | 41.6 | 0.88 (0.67-1.16) | 0.364 | 76.6 | 0.88 (0.54 –1.42) | 0.592 | 88.4 | 2.42 (0.93-6.32) | 0.072 |
| Separated, divorced, widowed | 51.5 | 1.02 (0.73-1.44) | 0.892 | 78.7 | 0.55 (0.28-1.07) | 0.079 | 76.5 | 0.64 (0.13-3.10) | 0.582 |
| **Occupational social grade** |  |  |  |  |  |  |  |  |  |
| DE (lowest social grade) (Ref) | 44.5 |  |  | 81.1 |  |  | 84.7 |  |  |
| C2 | 40.6 | 0.95 (0.70-1.29) | 0.740 | 77.2 | 0.74 (0.39-1.41) | 0.361 | 83.3 | 1.48 (0.41-5.39) | 0.549 |
| C1 | 47.8 | 1.28 (0.97-1.69) | 0.084 | 76.8 | 0.84 (0.49-1.44) | 0.519 | 84.0 | 1.16 (0.41-3.30) | 0.780 |
| AB (highest social grade) | 51.7 | 1.37 (1.03-1.82) | **0.031** | 81.2 | 1.08 (0.59-1.99) | 0.808 | 86.6 | 3.50 (0.95-12.99) | 0.061 |
| **Follow-up to a previous screening** |  |  |  |  |  |  |  |  |  |
| No (Ref) |  |  |  | 81.4 |  |  |  |  |  |
| Yes |  |  |  | 73.1 | 0.70 (0.44-1.11) | 0.132 |  |  |  |
| **Used a covid-19 infection self-test** |  |  |  |  |  |  |  |  |  |
| No (Ref) | 44.5 |  |  |  |  |  | 82.3 |  |  |
| Yes | 49.4 | 1.16 (0.95-1.42) | 0.142 |  |  |  | 90.8 | 0.42 (0.15-1.18) | 0.100 |
| **Used a bowel cancer screening self-test** |  |  |  |  |  |  |  |  |  |
| No (Ref) | 45.5 |  |  |  |  |  |  |  |  |
| Yes | 56.4 | 1.19 (0.85-1.65) | 0.316 |  |  |  |  |  |  |
| **Used a blood self-test** |  |  |  |  |  |  |  |  |  |
| No (Ref) | 45.5 |  |  | 77.6 |  |  |  |  |  |
| Yes | 51.4 | 1.38 (1.07-1.77) | **0.014** | 85.4 | 2.17 (1.17-4.03) | **0.014** |  |  |  |
| **Used a vaginal self-test** |  |  |  |  |  |  |  |  |  |
| No (Ref) | 47.5 |  |  | 79.9 |  |  |  |  |  |
| Yes | 39.9 | 0.78 (0.55-1.11) | 0.171 | 71.4 | 0.86 (0.42 –1.76) | 0.686 |  |  |  |

*Note: Only variables that were significant at p<.1 in univariable analysis or where an a-priori decision had been made to include them in the multivariable models are included in the table. Percentages are unadjusted. Ref: reference category. Nagelkerke pseudo-R² was 0.04 for regular attenders, 0.06 for irregular attenders and 0.17 for never attenders.*

**Table S3: Binary Logistic Regression exploring predictors of a don’t know response (compared to making a screening choice), stratified by previous screening attendance**

|  | ***Regular Attenders (n=2310)*** | | | ***Irregular Attenders (n=871)*** | | |
| --- | --- | --- | --- | --- | --- | --- |
|  | *% selecting don’t know response* | *AOR (95% CI)* | *p* | *% selecting don’t know response* | *AOR (95% CI)* | *p* |
| **Age (continuous)** |  | 1.00 (0.98-1.01) | 0.717 |  | 0.98 (0.95-1.01) | 0.157 |
| **Ethnicity** |  |  |  |  |  |  |
| Any White background *(Ref)* | 11.9 |  |  | 8.2 |  |  |
| Ethnic minority background | 9.1 | 0.85 (0.48-1.49) | 0.558 | 17.1 | 2.18 (1.02-4.67) | **0.044** |
| **Sexual Orientation** |  |  |  |  |  |  |
| Heterosexual *(Ref)* | 11.8 |  |  | 9.1 |  |  |
| Lesbian, Gay, Bisexual and Other (LGB) | 9.5 | 1.04 (0.51-2.14) | 0.913 | 10.9 | 1.55 (0.61-3.94) | 0.361 |
| **Marital Status** |  |  |  |  |  |  |
| In a relationship *(Ref)* | 12.3 |  |  | 8.2 |  |  |
| Single | 11.4 | 0.83 (0.56-1.25) | 0.378 | 8.8 | 0.78 (0.40-1.52) | 0.464 |
| Separated, divorced, widowed | 7.3 | 0.51 (0.27-0.95) | **0.033** | 14.4 | 2.65 (1.30-5.40) | **0.008** |
| **Occupational social grade** |  |  |  |  |  |  |
| DE (lowest social grade) *(Ref)* | 11.9 |  |  | 11.9 |  |  |
| C2 | 13.4 | 1.00 (0.66-1.52) | 0.992 | 7.5 | 0.69 (0.31-1.54) | 0.365 |
| C1 | 10.8 | 0.76 (0.50-1.14) | 0.181 | 8.2 | 0.59 (0.30-1.15) | 0.122 |
| AB (highest social grade) | 11.2 | 0.85 (0.56-1.27) | 0.416 | 7.9 | 0.61 (0.29-1.26) | 0.178 |
| **Used a urine self-test** |  |  |  |  |  |  |
| No (Ref) | 12.5 |  |  |  |  |  |
| Yes | 7.9 | 0.49 (0.31-0.79) | **0.003** |  |  |  |
| **Used a vaginal self-test** |  |  |  |  |  |  |
| No (Ref) | 12.1 |  |  |  |  |  |
| Yes | 8.4 | 0.87 (0.52-1.47) | 0.608 |  |  |  |

*Note: Only variables that were p<.1 in the univariable analysis or an a-priori decision had been made to include them in the multivariable models are shown in the table. The model for never attenders is not reported due to a small group size (n=26). Percentages are unadjusted. Ref: reference category. Nagelkerke pseudo-R² was 0.02 for regular attenders and 0.06 for irregular attenders.*

**Table S4: Coding of free-text responses for reasons self-sampling was chosen (n=701 participants)**

|  | **n** | **%** |
| --- | --- | --- |
| Women have previous negative experiences with clinicians or the screening procedure | 36 | 1.9% |
| Women prefer to have vaginal and non-speculum sampling | 34 | 1.8% |
| Other - cannot allocate to other themes | 33 | 1.8% |
| Self-sampling may save the NHS time or resources | 32 | 1.7% |
| Self-sampling helps those with physical disabilities or mental health issues | 20 | 1.1% |
| Self-sampling overcomes issues with booking a screening appointment | 20 | 1.1% |
| Self-sampling will help overcome competing priorities | 18 | 1.0.% |
| Self-sampling is less stressful than clinician screening | 14 | 0.7% |
| Self-sampling is less intrusive and more dignified | 13 | 0.7% |
| Self-sampling overcomes other covid-related reasons (excluding risk) | 13 | 0.7% |
| Women's choice of self-sampling was influenced by information in the survey | 13 | 0.7% |
| Self-sampling can be done in the comfort of women's own home | 11 | 0.6% |
| Self-sampling is preferred by women with a-typical anatomy | 10 | 0.5% |
| Women are confident in their ability to do self-sampling | 10 | 0.5% |
| Women have confidence in the results of a self-sampling test | 8 | 0.4% |
| Self-sampling is a quick procedure | 5 | 0.3% |
| Self-sampling is more attractive to those who have experienced physical/sexual abuse | 5 | 0.3% |
| Women discuss the need for a second test if they are HPV positive | 5 | 0.3% |
| Self-sampling feels like a safer screening option | 3 | 0.2% |
| Self-sampling is a novel screening option | 2 | 0.1% |

*Note:* Percentages are calculated as a proportion of the total number of participants that selected self-sampling (n=1,886)

**Table S5: Coding of free-text responses for reasons clinician screening was chosen (n=468 participants)**

|  | **n** | **%** |
| --- | --- | --- |
| Women lack confidence in their ability to do the self-sampling test | 55 | 4.1% |
| Screening is a job for professionals who know what they are doing | 27 | 2.0% |
| Clinician screening gives reassurance to those medically at-risk | 21 | 1.6% |
| Other - cannot allocate to other themes | 19 | 1.4% |
| Women do not think self-sampling will work for a-typical anatomy | 15 | 1.1% |
| Women feel more comfortable or relaxed with clinician screening | 13 | 1.0% |
| There is an opportunity to identify other issues during clinician screening | 10 | 0.8% |
| Individuals with health issues may struggle to carry out self-sampling | 10 | 0.8% |
| Clinician screening feels like a safer screening option | 8 | 0.6% |
| Women have misunderstood self-sampling or clinician screening | 7 | 0.5% |
| Women think it is easier to go to the doctor for screening | 6 | 0.5% |
| Women are anxious about the pain or intrusiveness of a self-sampling test | 3 | 0.2% |
| Women are more likely to attend screening if it is done by a clinician | 2 | 0.2% |

*Note:* Percentages are calculated as a proportion of the total number of participants that selected clinician screening (n=1,340)
